# The Anatomy of the Peripheral Nerves

**Published:** 1920-09

**Authors:** 


**The Anatomy of the Peripheral Nerves.** By A. Melville Paterson, M.D., F.R.C.S., Lieut.-Col., R.A.M.C. Pp. xi., 165. London: Oxford Medical Publications. 1919. Price 12s. 6d. net.

This book is designed for the use of military orthopædic surgeons and students, and gives a detailed and precise account of the peripheral nerves, as well as of the cranial and sympathetic. The diagrams are remarkably clear and numerous, one in fact to every two pages—many of them have been made from original dissections. We find interesting sections on the morphology and development of the nerves and on the great plexuses both of the spinal nerves and of the sympathetic. The book would be improved by a fuller account of the abnormalities frequently met with and the location in the trunks of the upper limb of the fibres to each group of muscles so far as they are known.

